# New Approach Based on Pix2Pix–YOLOv7 mmWave Radar for Target Detection and Classification †

**DOI:** 10.3390/s23239456

**Published:** 2023-11-28

**Authors:** Mohamed Lamane, Mohamed Tabaa, Abdessamad Klilou

**Affiliations:** 1 MiSET Team, Faculty of Sciences and Technics FST, Sultan Moulay Slimane University, Beni Mellal 23030, Morocco; com.lamane@gmail.com (M.L.); a.klilou@usms.ma (A.K.); 2 Pluridisciplinary Laboratory of Research & Innovation (LPRI), EMSI, Casablanca 23300, Morocco

**Keywords:** mmWave, radar, FMCW, classification, detection, YOLOv7, Pix2Pix

## Abstract

Frequency modulated continuous wave (FMCW) radar is increasingly used for various detection and classification applications in different fields, such as autonomous vehicles and mining fields. Our objective is to increase the classification accuracy of objects detected using millimeter-wave radar. We have developed an approach based on millimeter-wave radar. The proposed solution combines the use of an FMCW radar, a YOLOv7 model, and the Pix2Pix architecture. The latter architecture was used to reduce noise in the heatmaps. We create a dataset of 4125 heatmaps annotated with five different object classes. To evaluate the proposed approach, 14 different models were trained using the annotated heatmap dataset. In the initial experiment, we compared the models using metrics such as mean average precision (mAP), precision, and recall. The results showed that the proposed model of YOLOv7 (YOLOv7-PM) was the most efficient in terms of mAP_0.5, which reached 90.1%, and achieved a mAP_0.5:0.95 of 49.51%. In the second experiment, we compared the models with a cleaned dataset generated using the Pix2Pix architecture. As a result, we observed improved performances, with the Pix2Pix + YOLOv7-PM model achieving the best mAP_0.5, reaching 91.82%, and a mAP_0.5:0.95 of 52.59%.

## 1. Introduction

FMCW radar systems find widespread application due to their numerous advantages. It can be used in low-visibility conditions. FMCW radar can be used to measure the speed, distance, and angle of an object with high accuracy. Additionally, it has the capability to generate azimuth–range heatmaps, providing precise information about an object’s distance, angle, and even its micro-Doppler signature [1]. Some of the most common applications of FMCW radar include automotive radar systems for adaptive cruise control (ACC) [2] to measure the distance between the vehicle and the vehicle in front and adjust the speed of the vehicle accordingly to maintain a safe following distance. Furthermore, it plays a crucial role in blind spot detection (BSD) [3], enabling the radar to detect vehicles in the driver’s blind spot. This feature provides an essential warning to the driver when changing lanes, mitigating potential dangers. The FMCW radar can be used in unmanned aerial vehicles (UAVs) [4,5] for obstacle detection to ensure the safety of the UAV and prevent collisions [6]. FMCW radar systems are also widely used for range-finding in various industrial applications [7]. In the field of surveillance and security, FMCW radar systems are used for perimeter protection and intrusion detection [8]. This is particularly critical in scenarios where workers are in constant contact with heavy equipment such as trucks, crushers, or backhoes. The drivers of these machines do not have a good view of the working area. This makes the risk of accidents very relevant. Industrial fatal and non-fatal accidents represent 16%, while the construction sector represents 17%. In the mining sector, accidents account for only 1%, but more than 80% of these accidents are fatal [9]. The detection and classification of objects is becoming more and more important in many applications, such as advanced driver assistance systems (ADAS) for automobile or machinery, surveillance, and security in industrial environments that are relatively dangerous. Indeed, in some factories, we can find very dangerous areas for humans. In the event of the presence of an individual, an emergency stop must be triggered.

Advanced driver assistance systems (ADAS) are increasingly integrated into various vehicles, including construction machinery, cars, trucks, and more [10]. These systems are based on a variety of hardware and software architectures. They use several sensors and detection instruments to ensure redundancy, thus, increasing the reliability of the system [11]. Among the sensors that can be associated with ADAS, cameras are popular for their high accuracy and cost-effectiveness. They provide an image at each time interval that can be represented as a 3-D matrix if the image is in color. They can be used with most classification architectures [12,13,14]. Among the disadvantages of using a camera [15] are that it cannot work in bad visibility conditions such as bad weather, the presence of dust, or the absence of a light source. Another disadvantage is that it cannot measure the position of objects in space.

Radar-based solutions are becoming more and more interesting, especially the FMCW radar that works with millimeter waves (mmWave). This radar continuously sends out a radiofrequency (RF) signal, with a frequency that evolves like a ramp from a minimum to a maximum frequency [16], and then starts again. The more antennas the FMCW radar has at the receiving end, the higher the resolution and accuracy. However, most of the objects detected using FMCW radar have a micro-Doppler signature [17,18]. This makes the classification of objects possible. The FMCW radar gives good position measurements for objects in space. It is more powerful and accurate than the ultrasonic or infrared sensors used in automotive applications [19].

Our work consists of improving the detection and classification of targets in environments with poor visibility. We propose an architecture based on an FMCW radar and the deep-learning architectures YOLOV7 and Pix2Pix. Our proposed approach consists of retrieving data from the radar as heatmaps, then cleaning it with the Pix2Pix architecture, and finally performing the classification using the proposed model of YOLOv7 [20]. To compare our work, we trained 14 models from different architectures such as Faster RCNN [21], YOLOv3, YOLOv5 [22] and YOLOv7. Moreover, our proposed architecture optimizes computing power.

The major contributions of our work are:

- The proposal and implementation of a new architecture based on the mmWave-radar–YOLOv7–Pix2Pix.
- The creation of a dataset of 1510 heatmaps and making it available online [23].
- A comparison of classification results on cleaned and uncleaned datasets.
- A proposal for a new YOLOv7 model.

This paper is organized as follows. In Section 2, we present the related work. In Section 3, we present the materials and methods used in this work. We present the proposed architecture and the experimental setup based on the mmWave radar. We present the dataset created with our testbed. In Section 4, we describe the deep-learning architecture used in this work. We describe the YOLOv7 architecture and the Pix2Pix architecture. Finally, in Section 5, we present and discuss the results obtained. We compare the different models. We conclude in Section 6 with a summary.

## 2. Related Works

Several researchers have explored the application of image classification on FMCW radars. In this section, we will give a review of the literature covering the evolution and use of YOLO algorithms and the architecture in this field. In this paper [24], the authors present a real-time architecture for multi-class recognition. This system is dedicated to ADAS embedded in construction machines. Knowing that construction sites are very dusty environments means that cameras will not be efficient. This architecture is based on a mmWave–YOLO combination. It performed the classification of six classes of objects, with an execution time of 46.6 ms.

In [14], the authors utilized YOLOv3, an earlier iteration of the YOLO framework. Additionally, their dataset comprised 840 images, with 740 captured under good visibility conditions and 140 in scenarios of poor visibility. This dataset is too small to have statistically reliable results. In [25], the authors propose an algorithm for object detection and classification based on a fusion between the camera and the mmWave radar. The region of interest is computed from the position of the object detected using the camera and radar. Then they propose an architecture based on Faster RCNN [21]. They called it RCF-Faster RCNN. The dataset they used is made of time–frequency spectrogram images. This technique allows the classification of some objects, but it cannot give the position of the targets in space. In this work, the authors present a digital beamforming approach using an FMCW radar. They use micro-Doppler signatures to classify human movements such as walking, running, and jumping. Then they use these features to train a DNN on the classification of activities. They obtain good performance for their model.

In [26], the authors created a dataset consisting of range–azimuth images capturing objects detected using an FMCW radar. They then proceeded to perform object classification using the YOLOv3 architecture, which is based on the Darknet53, across different scenarios and target variations. The authors in [27] performed the simultaneous classification of targets using YOLOv3 in the context of automotive FMCW radar. They acquired data from a cascaded mmWave radar and converted it into a cartesian representation. The authors conducted a comparative analysis, pitting a conventional model against their proposed model. The comparison relied on measuring processing time in milliseconds (ms). However, it is important to note that using milliseconds is not a robust basis for comparison because it does not account for hardware variations. In embedded systems, like those in automotive applications, it is crucial to evaluate computing power in terms of BFLOPS. This approach is essential for developing a model that can effectively operate within the resource constraints of automotive systems, which often lack access to high-powered computing resources.

In [28], the authors developed a multi-algorithm architecture for the purposes of object detection and classification. This architecture utilizes two heatmap inputs. The first heatmap is based on range–azimuth–Doppler data, while the second heatmap is derived from range–velocity heatmap. For the classification tasks, they used YOLOv4 [29] for both of these inputs. Subsequently, a coordinate transformation was applied, converting data from the range–azimuth representation to the Cartesian representation, allowing for classification using the YOLOv4 model.

In paper [30], the authors present a technique for angle-of-arrival estimation using a mmWave radar with mechanical rotation. The rotation increases the azimuthal field-of-view. The proposed method estimates the angle-of-attack of targets using a single transmitter and receiver. Measurements are performed in several cases. The obtained data are used to create distance–angle heatmaps and morphological operators are used to estimate the angle of the objects.

In [31], the authors have implemented an architecture called MS–YOLO. It is a combination of camera and radar, with a multi-data source-object-detection network. They used YOLOv5 for classification. Their model gives good results. This paper [32] presents the feasibility and effectiveness of using low-noise microwave amplifiers integrated with a 24 GHz radar to detect targets. They embarked on training a machine-learning (ML) model, specifically a support vector machine (SVM), using the recorded data to categorize targets into four distinct classes. They obtain a good F1 score using the SVM method with an RFB kernel. They then compare their model with other algorithms.

In this paper [33], the authors propose a method to accelerate the convolutional neural network (CNN) on the field programmable gate array (FPGA) for embedded applications. Considering the micro-Doppler signature caused by object movements, the mmWave radar spectrogram is adopted as the input of the CNN. Comparisons and discussions are conducted on the measured data. This paper [34] presents a review of various deep-learning approaches for radar to accomplish some important tasks in an autonomous driving application, such as detection and classification. The authors have classified the paper according to the different methods and techniques used. This is one of the critical aspects of using radar data with deep-learning models. They review recent multi-sensor-fusion models (radar, camera, lidar, etc.) based on deep learning.

In this paper [35], the authors present a method for object classification based on machine learning and extract fast Fourier transform (FFT) features using millimeter-wave FMCW radars. Measurements are performed for several real-world situations. They used several machine-learning algorithms, such as support vector machine (SVM), naive Bayes, gradient boosting (GBM), and logistic regression. Our work [36] focuses on the classification of heatmaps obtained from the Coloradar dataset of mmWave cascade radar [37]. The architecture used to perform the classification is YOLOv5. We compared several models to determine which one is best suited for an embedded system.

In [38], the authors introduce a millimeter-wave radar cane, enhancing mobility for visually impaired individuals. By integrating a 122 GHz radar into a traditional white cane, it detects obstacles and distinguishes humans from non-human entities based on their vital signs. The technology showcases promise for improved mobility and safety for the visually impaired.

In this work, we obtained a mAP_0.5 of 91.82% for the association of Pix2Pix with YOLOv7-PM. This result is better than in [24], where the authors obtained a mAP_0.5 of 72% using YOLOv3.

In [25], the authors obtained 89.45%, which is still lower than the results obtained. The result in [26] is 97.6%, an abnormally high mAP_0.5. Our results are close to those in [27]. However, our results are significantly better than those in [28,31,36]. We cannot compare our results with [30,32,35] as the authors do not use the metrics used in image classification.

Table 1 compares the different works based on applications, equipment, methods, and results.

## 3. Materials and Methods

### 3.1. FMCW Radar Overview

**Figure 1:**
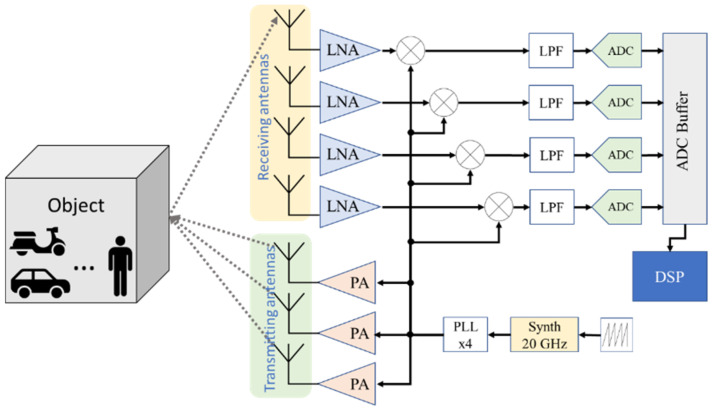
FMCW radar front-end schematic.

Frequency modulated continuous wave (FMCW) radars gradually vary the frequency of their signal by ascending ramps. In this method, a synthesizer generates a signal with a variable frequency that varies as a sawtooth function. This signal is amplified using a power amplifier (PA) and transmitted using one or more antennas [39,40]. When a signal is reflected by an object, it is received by the receiving antennas and then amplified using a low-noise amplifier (LNA). An intermediate frequency (IF) is obtained using the product of the transmitted signal and the received signal, which is then filtered using a low-pass filter (LPF). An analog-to-digital converter (ADC) samples the IF signal, then stores it on a buffer [16]. A digital signal processor (DSP) recovers the contents of the buffer to apply the signal-processing algorithms (1D-FFT, 2D-FFT, CFAR, etc.). Figure 1 shows the simplified schematic of the FMCW radar.

The transmitted signal is defined in (1):

(1) $$S_{Tx}\left(t\right)=A_{1}\sin\left(\omega_{1}(t)t+\varphi_{1}(t)\right)$$

where $\omega_{1}\left(t\right)=2\pi f_{1}\left(t\right)$ and $f_{1}(t)$ is the transmitted frequency, $A_{1}$ is the amplitude of the transmitted signal, and $\varphi_{1}\left(t\right)$ is the phase of the transmitted signal.

The received signal is defined in (2):

(2) $$S_{Rx}\left(t\right)=A_{2}\sin\left(\omega_{2}(t)t+\varphi_{2}(t)\right)$$

where $\omega_{2}\left(t\right)=2\pi f_{2}(t)$ and $f_{2}(t)$ is the received frequency, $A_{2}$ is the amplitude of the received signal, and $\varphi_{2}\left(t\right)$ is the phase of the received signal.

The two signals are mixed using a mixer, and we obtain (3):

(3) $$S\left(t\right)=\frac{1}{2}A_{1}A_{2}\left(\cos\left(\left(\omega_{1}\left(t\right)-\omega_{2}\left(t\right)\right)t+\left(\varphi_{1}\left(t\right)-\varphi_{2}\left(t\right)\right)\right)\right.\;\left.\;\;\;\;\;\;\;\;\;\;\;\;\;\;\;\;\;\;\;\;\;\;\;\;\;\;\;\;\;+\cos\left(\left(\omega_{1}\left(t\right)+\omega_{2}\left(t\right)\right)t+\left(\varphi_{1}\left(t\right)+\varphi_{2}\left(t\right)\right)\right)\right)\;$$

Passing the signal through a low-pass filter, we obtain (4):

(4) $$S\left(t\right)=\frac{1}{2}A_{1}A_{2}\cos\left(\left(\omega_{1}\left(t\right)-\omega_{2}\left(t\right)\right)t+\left(\varphi_{1}\left(t\right)-\varphi_{2}\left(t\right)\right)\right)$$

By applying the FFT on the function (4), we can determine the frequency variation (5):

(5) $$∆f=\frac{\omega_{1}\left(t\right)-\omega_{2}\left(t\right)}{2\pi }$$

By using (5), the distance can be calculated using the following Equation (6):

(6) $$R=\frac{cT_{chirp}∆f}{2B}$$

where, *c* is the celerity of light, *T_chirp_* is the time to pass from the minimum frequency to the maximum frequency, and *B* is the bandwidth.

**Figure 2:**
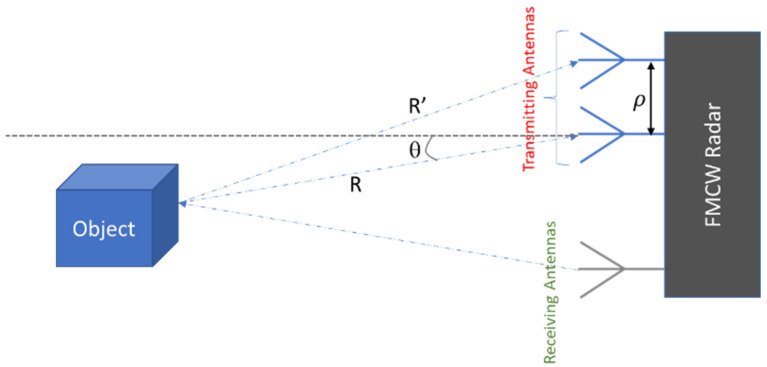
Illustration of the angle of an object with regard to a FMCW radar.

In order to determine the angle of the object relative to the radar, at least two antennas are needed in reception. Figure 2 shows an illustration of the angle of an object relative to an FMCW radar. The first antenna measures a distance *R*; it is assumed that the object is sufficiently far to say that the second antenna will measure a distance $R^{\prime }\approx R+\rho \sin\theta$, with *ρ* being the distance between two receiving antennas, and *θ* being the angle we are trying to determine.

Equation (8) gives the expression for *θ*.

(7) $$\theta ={sin}^{-1}\left(\frac{\left(R^{\prime }-R\right)}{\rho }\right)$$

If we can determine *R* and *θ*, we can transform from a polar representation to a Cartesian one:

(8) $$\left(\begin{array}{c} x\\ y \end{array}\right)=\left(\begin{array}{c} R\cos\left(\theta \right)\\ Rsin\left(\theta \right) \end{array}\right)$$

The example we have chosen is a simplification of the working of an FMCW radar, because we have one antenna in transmission and two in reception. In fact, the radar we used has three antennas in transmission and four in reception. The more antennas in reception, the better the angular resolution.

### 3.2. The Proposed Architecture

**Figure 3:**
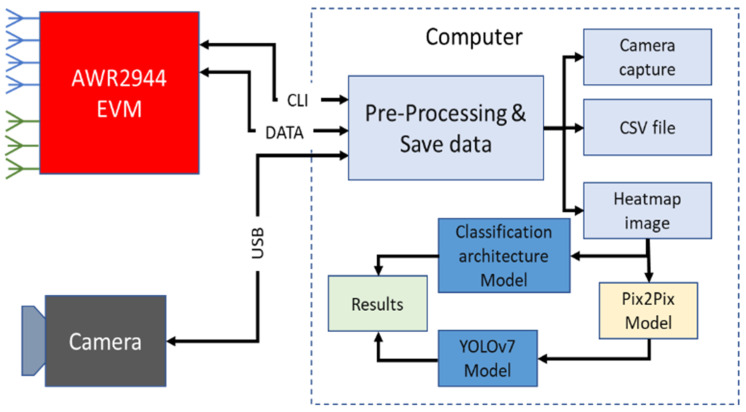
Architecture of work.

First, we will outline the methodology we employed, as shown in Figure 3. Our first step was to acquire radar data in CSV format. Each file contained a 100 × 100 matrix, effectively representing a heat map and constituting our dataset. The second step was to annotate all the images using Roboflow software (Available online: https://roboflow.com/). For each image in the dataset, a corresponding .txt file was generated to store the annotations. It contains the coordinates of the bounding boxes of each object on it. To verify the effectiveness of our proposed method, we proceeded in two different ways. The first involved training YOLOv7 directly, without processing the images. The second was to process the images using the Pix2Pix architecture. Then, we tested YOLOv7. For a reliable comparison, we trained six other models for other architectures such as Faster RCNN, YOLOv3, and YOLOv5. We compared the results to see which method is more efficient.

### 3.3. AWR2944 Radar Overview

The use of a millimeter-wave FMCW radar in this dataset creation process provides several advantages over other sensing technologies. The mmWave radar operates in a frequency range that is less crowded than other frequency bands, which reduces the likelihood of interference from other devices. It also has a relatively high resolution, which allows for accurate detection and localization of objects. The AWR2944 evaluation board [41] is an evaluation platform that enables users to evaluate and demonstrate the capabilities of the AWR2944 device, which is a highly integrated, single-chip mmWave sensor for automotive radar applications.

The evaluation is equipped with essential features and components that facilitate users in testing and evaluating the performance of the AWR2944 device, including a fully integrated mmWave sensor based on the AWR2944 chip, which includes a 76 GHz to 81 GHz frequency-band transceiver, a digital-signal processor, and a microcontroller. An integrated control-and-communication interface allows users to connect to and control the device via a USB or UART interface. A power supply circuit provides the necessary voltage and current to power the device, and includes a power switch, a power-indicator LED, and an on-board voltage regulator. On-board signal-processing and data-storage capabilities, which include DDR memory and flash memory, connectors, and interfaces that enable users to connect the board to external equipment such as JTAG, debug, UART, USB, and I2C interfaces. An integrated PCB antenna is also provided on the evaluation board. The evaluation board is designed to support the evaluation and development of various radar-based applications. It supports various modes of operation, including chirp- and frame-based operation, and provides a wide range of configuration options for the transceiver and signal-processing chain. The evaluation board is compatible with TI’s mmWave Studio software (version 4.2.0), which provides a graphical user interface for configuring, controlling, and analyzing the performance of the AWR2944 device.

### 3.4. Experimental Setup

**Figure 4:**
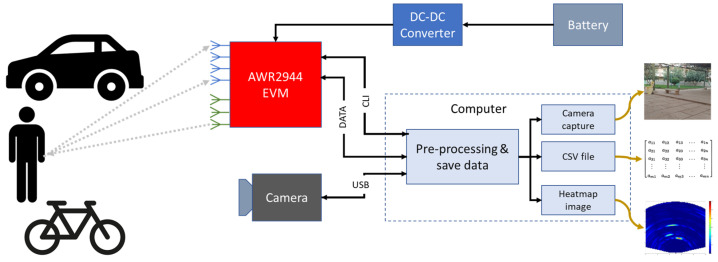
Architecture of experimental setup.

In order to create our dataset for detection and classification of objects, a hardware and software architecture is implemented based on the AWR2944 [42] evaluation board. It works by transmitting a continuous wave on three antennas and measuring the reflected signal on four antennas, which can then be used to determine the range, velocity, and angle of an object. The radar communicates with a computer through two UART (universal asynchronous receiver–transmitter) ports. The first UART port is used as a command-line interface (CLI) and is mainly used to send the front-end configuration to the radar. This allows for flexible control of the radar’s parameters, such as frequency, bandwidth, slope, and more. The second UART port is used to retrieve the raw data from the radar, which can then be processed and analyzed to detect and locate objects. The system’s portability and battery-powered operation make it suitable for use in outdoor settings. A camera is added to the system to help with annotation during the dataset creation process. Figure 4 shows the proposed architecture of the experimental setup.

**Figure 5:**
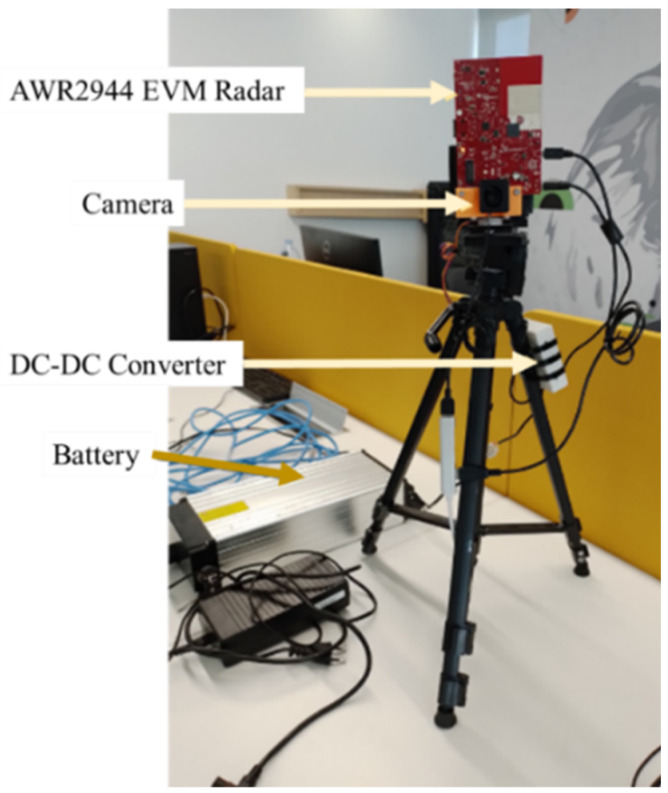
View of the experimental setup.

The camera captures images of the environment, and the objects within it, that can be used as a reference for the radar data. A program has been developed to record the data from the radar and camera in synchronization. Figure 5 is a view of the experimental setup.

This enables a precise correlation between the radar data and the corresponding camera image. The program can capture 4 frames per second, which, in terms of radar data, is considered a relatively low frame rate. The dataset created with this hardware and software architecture is useful for object detection in various scenarios. It can be used for autonomous vehicles, drones, security systems, and more. The radar data provides information about the distance, velocity, and angle of objects, which can be used to detect and locate them in real-time. The camera images provide additional information such as color, shape, and texture, which can be exploited to improve the accuracy of object detection. In addition to object detection, the dataset can also be used for applications like object tracking, motion analysis, and environment mapping. The camera images can be used to create a map of the environment, which can be useful for navigation and localization.

## 4. Deep-Learning Architectures

### 4.1. YOLOv7 Architecture

YOLOv7 (You Only Look Once, version 7) is a real-time object-detection system proposed by [20]. It is an improvement over previous versions of YOLO and is one of the fastest and most accurate object-detection systems currently available. Built upon a convolutional neural network (CNN) architecture, the YOLOv7 system employs a neural network to predict multiple bounding boxes and class probabilities for objects in an image. The system is able to process an image in real-time, making it suitable for use in a wide range of applications such as self-driving cars, surveillance, and robotics. One of the key features of YOLOv7 is its use of anchor boxes. Anchor boxes are predefined bounding boxes that are used to anchor the CNN’s predictions for objects in the image. These anchor boxes are chosen to be the most representative of objects in the training dataset and help to improve the accuracy of the object-detection system. Another important feature of YOLOv7 is its use of a multi-scale prediction strategy. The system uses a single neural network to predict objects at multiple scales, which helps to improve the detection of small objects in an image. The system also uses a technique called “feature pyramid networks” to combine features from different layers of the CNN, which helps to improve the accuracy of the object-detection system. It has achieved state-of-the-art results on several benchmark datasets, such as the COCO dataset, and has been shown to be able to detect a wide range of objects in real-time. Overall, YOLOv7 is a highly advanced object-detection system that is suitable for use in a wide range of applications.

**Figure 6:**
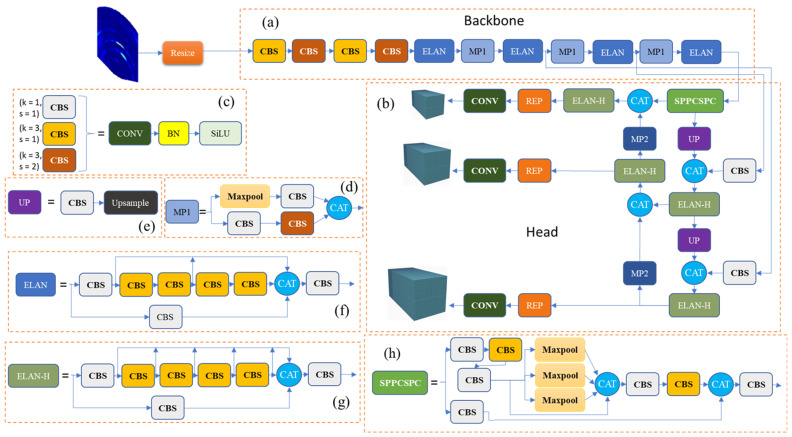
Architecture of YOLOv7: (**a**) backbone, (**b**) head, (**c**) CBS, (**d**) MP1, (**e**) UP, (**f**) ELAN, (**g**) ELAN-H, and (**h**) SPPCSPC.

We analyzed YOLOv7 as a whole. First, the input image is resized to 1240 × 1240 × 3 or 640 × 640 × 3, depending on the models used. Then, the image is fed into the backbone network. The network comprises CBS, which in turn consists of convolution (CONV), batch normalization (BN), and sigmoid linear unit (SiLU) layers. Refer to Figure 6 for a visual representation. After passing through four CBS, we pass our element through an ELAN block. The ELAN module consists of a number of CBS where the size of the input and output remains the same; Figure 6f shows its composition. Next comes the MP layer, which is divided into CBS and Maxpool, where the difference between MP1 and MP2 is a change in the ratio of the number of channels; Figure 6d shows its composition. The outputs of blocks MP1 + ELAN correspond to the outputs C3, C4, and C5. The head of YOLOv7 is a PAFPN structure [43]. The outputs C3, C4, and C5 are the inputs of the head. The head network consists of the SPPCSPC, Upsample, CBS, MP2, the concatenation module (CAT) and three subsequent REP modules. The SPPCSPC is similar to the SPPF used in the YOLOv5 architecture to increase the receptive field of a network. It has mainly the same structure as YOLOv5, but the CSP module used in YOLOv5 is replaced by the ELAN-H module. The ELAN-H module is slightly different from the ELAN module; it differs only in the number of CATs.

The precision is a metric that gives a positive true prediction percentage for all positive cases; it is given by the following equation:

(9) $$Precision=\frac{TP}{TP+FP}$$

The recall metric is calculated as the ratio of correctly predicted positive cases to all cases in the actual positive class. It is calculated as follows:

(10) $$Recall=\frac{TP}{TP+FN}$$

TP, FP, and FN represent true positive, false positive, and false negative, respectively. Average precision (AP) is a metric used to evaluate the performance of object-detection and image-retrieval systems. It is defined as the average of the precision values at each point where a positive detection is made. This considers both the number of true positives (correct detections) and false positives (incorrect detections) at each point. The precision at each point is calculated as the number of true positives divided by the number of true positives plus false positives. AP is a widely used metric for object-detection and image-retrieval tasks. It evaluates both the number of correct detections and the quality of those detections.

The mean average precision (mAP) is a commonly used evaluation metric in the field of object detection. It assesses both the accuracy of locating objects (through bounding box coordinates) and classifying them into categories. Many object-detection algorithms, such as Faster RCNN, SSD, YOLOv5, and YOLOv7, employ mAP as a metric to estimate the overall effectiveness and performance of their models.

The value of *mAP* is given by Equation (11):

(11) $$mAP=\frac{1}{N}\sum_{i=1}^{N}{AP}_{i}=\frac{1}{N}\sum_{i=1}^{N}\left(\int_{0}^{1}P_{i}\left(R\right)dR\right)$$

where *N* is the number of classes.

### 4.2. Pix2Pix

**Figure 7:**
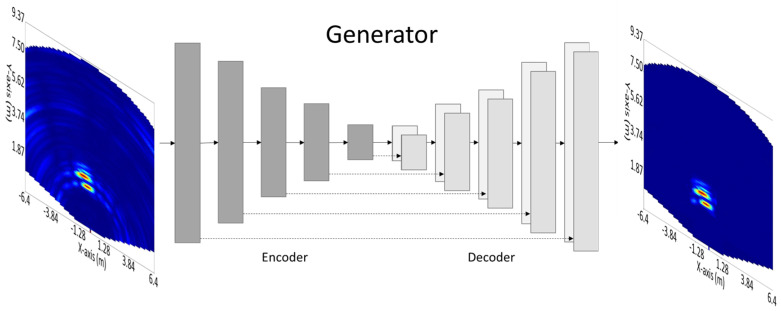
Architecture of the Pix2Pix generator model.

Pix2Pix [44] is a generative model that is trained to learn the mapping from an input image to an output image. It is particularly useful for image-to-image translation tasks, where the goal is to generate a new image that is similar to a given input image, but with certain modifications. One of the key features of Pix2Pix is that it uses a type of neural network called a conditional generative adversarial network (cGAN). A cGAN consists of two main components: a generator and a discriminator. The generator is responsible for generating new images (Figure 7 shows the architecture of the generator), while the discriminator is responsible for determining whether an image is real or fake. During training, the generator and discriminator are trained in an adversarial manner, with the generator trying to create images that can fool the discriminator, and the discriminator trying to accurately identify whether an image is real or fake. This process continues until the generator can create images that are indistinguishable from real images.

Pix2Pix is trained on a dataset of pairs of images, where the goal is to learn the mapping from the input image to the output image. The input image is typically a sketch or a low-resolution image, while the output image is a high-resolution version of the same scene. Once the model is trained, it can be used to generate new images by providing it with an input image.

The model will then use the mapping it learned during training to generate an output image that is like the input image but with certain modifications. One of the key advantages of Pix2Pix is that it can generate high-quality images that are similar to the input image. This makes it particularly useful for tasks such as image super-resolution, where the goal is to generate a high-resolution version of a low-resolution image. Pix2Pix can also be used for a wide range of other image-to-image translation tasks, such as converting sketches to photos, converting day to night, or converting black and white images to color. Another advantage of Pix2Pix is that it can generate images that are consistent with the input image. This is because the model is trained on a dataset of pairs of images and can learn the mapping from the input image to the output image.

**Figure 8:**
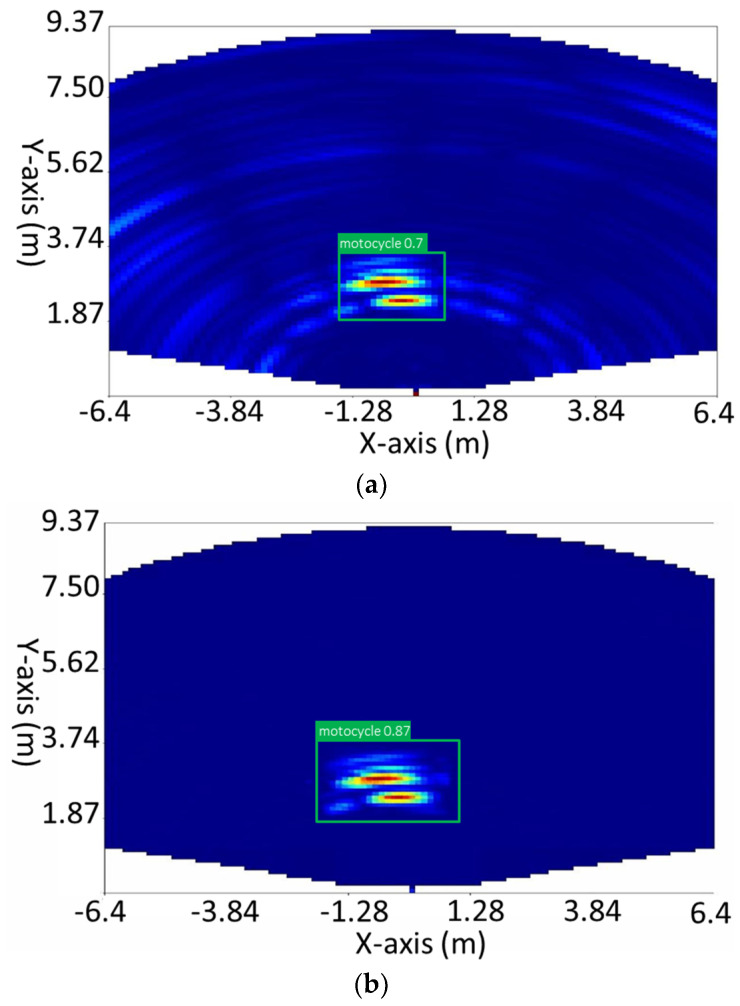
(**a**) Original image. (**b**) Image cleaned by Pix2Pix.

Overall, Pix2Pix is a powerful and versatile model that can be used for a wide range of image-to-image translation tasks. It can generate high-quality images that are similar to the input image and is able to generate images that are consistent with the input image. Due to its ability to generate high-quality images and its versatility, Pix2Pix has been widely used in various applications such as image editing, the video game industry, and virtual reality. Figure 8 shows an example before and after cleaning the heatmap using Pix2Pix.

## 5. Results and Discussions

### 5.1. Proposed Model

To create our YOLOv7-PM model, we took inspiration from the YOLOv7-X model, renowned for its speed and high performance. Our main objective was to optimize its performance by making significant adjustments at different levels of the model. This optimization process was iterative, involving several trials to arrive at the best possible parameters, particularly concerning anchors, input size, and the replacement of a key element. First, we decided to modify the input size of the model in relation to that of the base model, YOLOv7-X. This modification is crucial, as it affects the resolution of the input images and, consequently, the model’s ability to detect and locate objects accurately.

Secondly, we opted to replace a component called E-ELAN with an ELAN. Given that the YOLOv7-X model uses E-ELAN, this change indicates our desire to adapt the model to our specific needs. The choice of this replacement is essential, as the model’s components have different characteristics that influence performance.

Another crucial change was the adjustment of the anchor parameters. Anchors are essential reference points for object detection. By optimizing these parameters, we sought to improve the quality of object detection and the accuracy of their localization.

Our iterative approach to customizing the YOLOv7-PM model was essential to achieving the best possible results. Each iteration involved careful fine-tuning, rigorous testing, and benchmarking to select the most effective parameters. This iterative methodology enabled us to progressively improve our model.

### 5.2. Proposed Dataset

In this work, we utilized a dataset that was recorded with the AWR2944 [40], a second-generation single-chip radar. We have developed a program to retrieve 4 frames per second from the radar, and for each frame, a camera capture was taken. The received data were then transformed into a cartesian representation and recorded as a heatmap. This heatmap enables us to detect and classify various targets, such as humans, bicycles, motorcycles, cars, and walls.

Table 2 presents the configuration of the radar used during the recording of the dataset. We recorded more than 4125 heatmaps for different objects. These images were then annotated using Roboflow, which allowed us to generate datasets that are compatible with YOLOv7. The use of the AWR2944 radar in combination with a camera allows for a comprehensive understanding of the environment being recorded. The cartesian representation of the data obtained from the radar enables more accurate detection and classification of targets. The annotation of the images using Roboflow ensures compatibility with YOLOv7, which is a widely used object-detection model. Overall, this dataset provides a valuable resource for further research and development in the field of object detection and classification. We have made available 1510 annotated frames for YOLOv3, YOLOv5 and YOLOv7 [23].

### 5.3. Experimental Environment and Models Training

In our model-training process, we used a Tesla V100-SXM2-16GB graphics card and 51 GB of RAM. We trained 14 different models using various architectures, each for 300 epochs, for a total training time of 73 h and 35 min. One of these models is our YOLOv7-PM, which produced accurate predictions. Figure 8 shows some predictions from the YOLOv7-PM model. This model was created from the YOLOv7-X for object detection in heatmaps, by changing the layers of its architecture.

### 5.4. Results, Analysis, and Comparison

In this work, we are interested in improving the classification of targets detected using a mmWave radar. A dataset of 4125 heatmaps was created, and all objects on the heatmaps were annotated, containing 5 different classes. We used the Pix2Pix architecture to transform the noised heatmaps into cleaner heatmaps, so we created a new dataset consisting of noiseless heatmaps. Then we followed two different methods. The first method is to train the YOLOv7 models as well as other architectures such as Faster RCNN, YOLOX, and YOLOv5, with the noised dataset. We have trained four YOLOv7 models (YOLOv7-X, YOLOv7-PM, YOLOv7-W6, and YOLOv7-E6E). Our proposed model, YOLOv7-PM, is a customized version of YOLOv7-X. The developer of the architecture put forward the other models [25]. In the second approach, we used a cleaned Pix2Pix dataset that had been trained with a dataset of over 1200 images. We then re-trained the models, namely Pix2Pix + YOLOv7-X, Pix2Pix + YOLOv7-PM, Pix2Pix + YOLOv7-W6, and Pix2Pix + YOLOv7-E6E. We then compare the mAP_0.5, mAP_0.5:0.95, precision, and recall of the models; these metrics will allow us to compare the models accurately.

**Figure 9:**
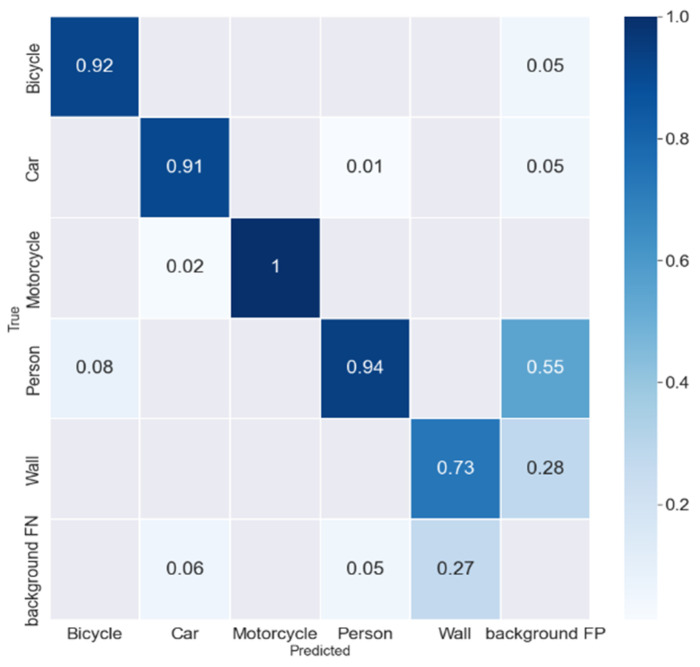
Confusion matrix for the YOLOv7-PM model.

DL models can sometimes confuse two categories; to illustrate this confusion we used a confusion matrix. This is a table used to evaluate the predictions of a model by comparing them to the real results. It has four components: true positives, true negatives, false positives, and false negatives. While precision is a basic measure of performance, precision and recall can also be calculated from the confusion matrix to evaluate the performance of a model. The confusion matrix is widely used in binary classification problems and can be extended to multi-class problems. It is an important tool for identifying areas of improvement and making informed decisions to improve the performance of a model. Figure 9 shows the confusion matrix for the YOLOv7-PM model.

**Figure 10:**
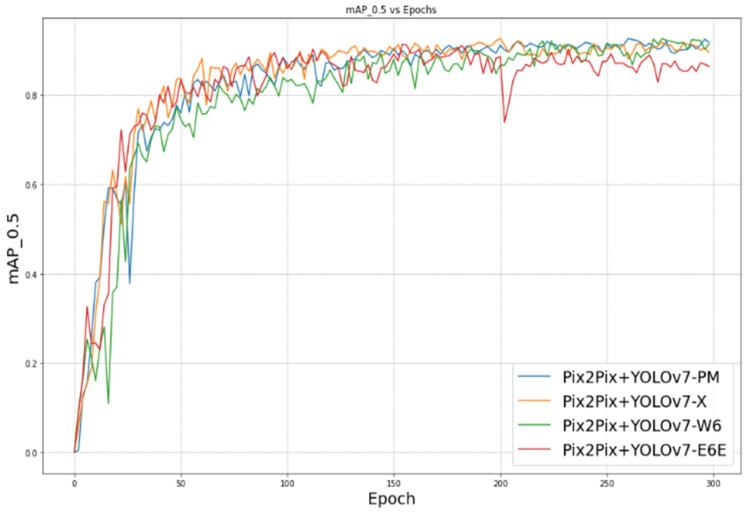
mAP_0.5 vs. epoch.

The mAP_0.5, also known as mean average precision at an intersection over union (IoU) threshold of 0.5, is a metric used to evaluate the performance of object-detection models. IoU measures the overlap between the predicted bounding box and the ground truth bounding box of an object in an image. Figure 10 shows the evolution of mAP_0.5 throughout the epochs.

We can see that during the first 50 epochs, the models Pix2Pix + YOLOv7-X and Pix2Pix + YOLOv7-E6E both surpass an accuracy of 83%. We can also notice that Pix2Pix + YOLOv7-E6E is an unstable model compared to the other models.

**Figure 11:**
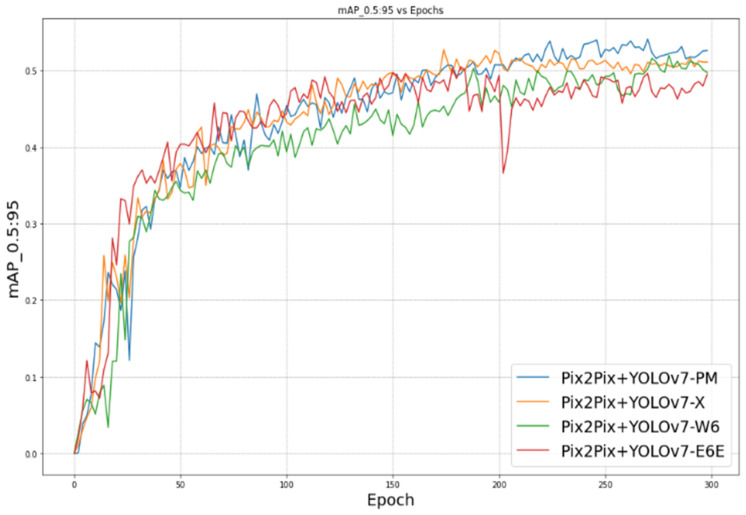
mAP_0.5:0.95 vs. epoch.

Figure 11 shows the evolution of mAP_0.5:0.95 during 300 epochs. The mAP_0.5:0.95, also known as mean average precision at IoU thresholds ranging from 0.5 to 0.95, is a metric used to evaluate the performance of object-detection models.

In classification algorithms, precision and recall are two widely used metrics for assessing the performance of a model. Precision measures the proportion of true positives (TP) out of all positive predictions (TP + false positives (FP)). In simpler terms, precision shows how accurately the model identifies positive instances among its predictions. A high precision score indicates that the model has a low false-positive rate, meaning it can accurately identify positive instances without many false positives.

**Figure 12:**
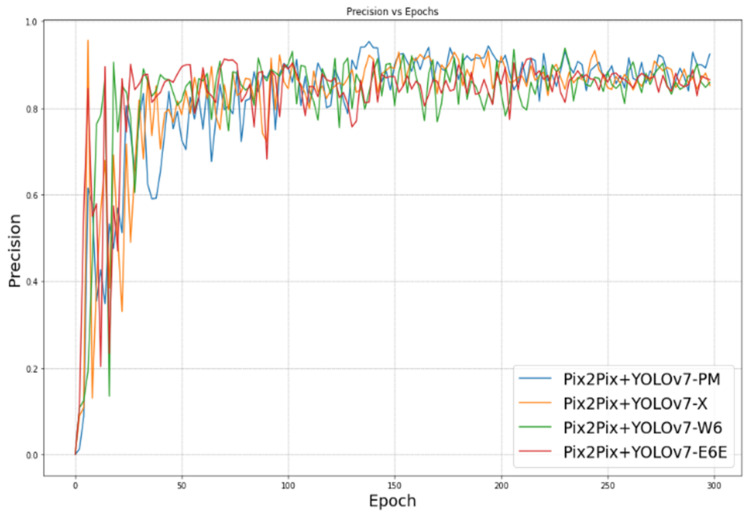
Precision vs. epoch.

**Figure 13:**
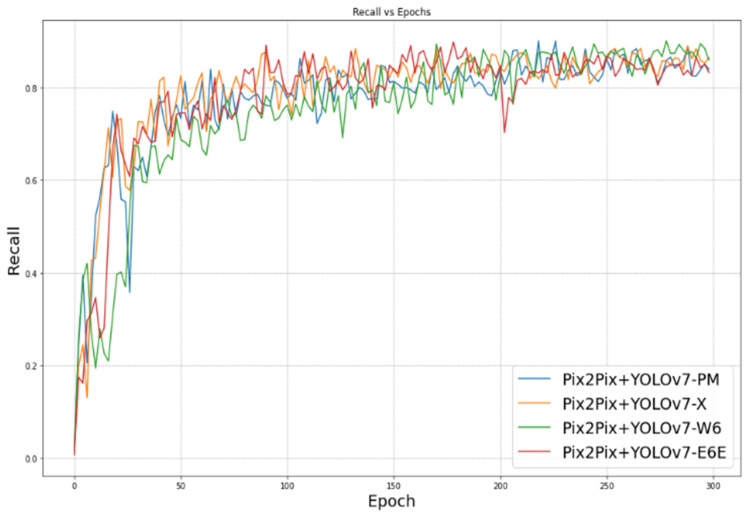
Recall vs. epoch.

Recall measures the proportion of true positives (TP) out of all actual positive instances (TP + False Negatives (FN)). Recall indicates how many of the actual positive instances the model is able to correctly identify. A high recall score means that the model has a low false-negative rate, indicating it can correctly identify most of the actual positive instances without many false negatives. The evolution of precision and recall during training can be observed in Figure 12 and Figure 13, respectively.

Training on the noised dataset

The first method is to train the models on the dataset that is not cleaned. The YOLOv7 models show good performances compared to the other models. The YOLOv7-PM model shows a mAP_0.5 of 90.1% and a mAP_0.5:0.95 of 49.51%, which are very good results compared to the other models trained on the uncleaned dataset. It also consumes 189 BFLOPs, which makes it an efficient model in terms of computing.

Training on the cleaned dataset

The second method uses the cleaned Pix2Pix dataset. Over 1200 images were used to train this architecture. We then re-train the same models (Pix2Pix + YOLOv7-X, Pix2Pix + YOLOv7-PM, Pix2Pix + YOLOv7-W6, and Pix2Pix + YOLOv7-E6E). We have seen a significant improvement in the performance of our model, with a mAP_0.5 of 91.82% and a mAP_0.5:0.95 of 52.59%. All this was achieved with a power consumption of 231.2 BFLOPs, which represents the best performance compared with other models. In terms of accuracy and computational efficiency, our model stands out as the best performing of the trained YOLOv7 models, placing it at the top in terms of computational efficiency. Table 3 shows the latest values of the main parameters. The second approach exploits the Pix2Pix dataset, which has been carefully prepared and trained with a set of over 1200 images.

Our proposed approach leans more towards being data-driven rather than model-driven. It demonstrates that improvements in the data can lead to effective performance even with simpler models. Figure 10, Figure 11, Figure 12 and Figure 13 illustrate the evolution of the four mentioned metrics over 300 training epochs. These graphs are smoothed and allow us to follow the progression of the models and see which model trains better than the others.

## 6. Conclusions

In this work, we have presented a method aimed at enhancing the accuracy of classifying targets detected using a mmWave radar. This method combines the use of a mmWave radar, the YOLOv7 architecture, and the Pix2Pix architecture. Then, we proposed a YOLOv7-PM model, which is an improvement of YOLOv7-X. We trained 14 different classification models using the annotated heatmap dataset and compared them with the conventional method without dataset cleaning, which gave us good results for the same models. In the first experiment, the models were compared using metrics such as mean accuracy (mAP), precision, and recall. The results showed that YOLOv7-PM was the most efficient model in terms of mAP_0.5, which reached 90.1%, and achieved a mAP_0.5:0.95 of 49.51%. In a follow-up experiment, the same models underwent retraining using a Pix2Pix architecture-generated clean dataset. This resulted in improved performance, with the Pix2Pix + YOLOv7-PM model achieving the best mAP_0.5 with 91.82% and a mAP_0.5:0.95 of 52.59%. This opens up the possibility of testing and comparing other preprocessing methods for our heatmap radar images. Our model, for which we see an improvement in the metrics, gives us a perspective to optimize Pix2Pix and to test and compare other architectures such as the cycleGAN. Then, we can compare our model with classification architectures such as faster RCNN, SSD, YOLOv3, and YOLOv5.

## Figures and Tables

**Table 1:** Comparative table.

References	Applications	Equipment	Methods	Results
[24]	Embedded ADAS for construction machines (real-time architecture for multi-class recognition).	- AGX-Xavier (Nvidia) - mmWave Radar RCS11 (Keycom Corp.)	YOLOv3 for object classification (six classes of objects).	mAP_0.5 = 0.72
[25]	Object detection and classification.	- Webcam DS-IPC-B12V2-I - mmWave radar MR3003	Camera and radar fusion. New architecture based on Faster RCNN (called RCF-Faster RCNN).	mAP_0.5 = 89.45% mAP_0.75 = 85.1%
[26]	Target classification using mmWave FMCW radars	- mmWave radar AWR2243	Object classification using YOLOv3 architecture based on Darknet53.	mAP_0.5 = 97.6%
[27]	Simultaneous classification of targets.	- mmWave radar	Simultaneous classification of targets based on YOLOv3 for an automotive FMCW radar.	mAP_0.5 = 92.07%
[28]	Detection and classification of two heatmaps inputs.	-AWR1843-BOOST - DFK 33UX273	Classification using the YOLOv4 on two inputs. The first is the range–azimuth–Doppler heatmap. The second is the range–velocity heatmap.	mAP_0.5 = 51.6%
[30]	Estimation of angle-of-arrival, using a mmWave radar with mechanical rotation.	- AWR1843EVM - Raspberry Pi 3 - PiCamera V2	Estimation of the angle-of-attack of targets using a single transmitter and receiver (1TX-1RX). Rotation increases the field-of-view in azimuth.	RMSE = 0.933
[31]	Fusion of camera and radar for classification.	- ARS 408-21 - Camera	Multi-data-source object-detection network. YOLOv5 used for classification.	mAP_0.5 = 84.1%
[32]	Feasibility and effectiveness of using low-noise microwave amplifiers integrated with a 24 GHz radar.	- mmWave radar - SVM	Machine-learning (ML) model called support vector machine (SVM) trained to classify the targets for 4 categories.	Accuracy = 96.32% F1-score = 96.28%
[35]	Classification based on machine learning and extracted fast Fourier transform (FFT).	- mmWave radar	Use of machine-learning algorithms such as support vector machine (SVM), naive Bayes, gradient boosting (GBM), and logistic regression for classification.	Accuracy = 95.6%
[36]	Classification of heatmaps obtained from mmWave cascade radar [37].	- Cascaded radar	Classification using YOLOv5. Comparison of several models to determine which one is best suited for an embedded system.	mAP_0.5 = 89.63%

**Table 2:** Radar parameters.

	Parameters
Framerate	4 Hz
Frequency	77 GHz
Waveform	FMCW
TX antennas	3
RX antennas	4
Range resolution	0.0732 m
Max range	9.3743 m
Azimuth resolution	14.5°
Power consumption	9 W
Data rate	<1 Mbps

**Table 3:** Training results.

Model	Params	Precision	Recall	mAP_0.5	mAP_0.5:0.95	BFLOPs
F-RCNN-R101-FPN+	60 M	0.8214	0.809	0.828	0.446	246
YOLOX-X [45]	99.1 M	0.8651	0.8752	0.8812	0.481	281.2
YOLOv5-N [22]	1.9 M	0.871	0.851	0.7541	0.4274	4.5
YOLOv5-M [22]	20.8 M	0.8863	0.867	0.8786	0.4902	47.9
YOLOv5-X [22]	86.2 M	0.8904	0.8261	0.8775	0.452	203.8
YOLOv5-L [22]	46.1 M	0.88	0.8543	0.8847	0.497	107.7
YOLOv7-X [20]	71.3 M	0.8174	0.8605	0.8531	0.4891	189
Pix2Pix + YOLOv7-X	92.1 M	0.8521	0.8643	0.896	0.5108	231.2
**Improvement**	**−20.8 M**	**+3.47%**	**+0.38%**	**+4.29%**	**+2.17%**	**−42.2**
YOLOv7-PM	71.5 M	0.8923	0.8412	0.901	0.4951	189
Pix2Pix + YOLOv7-PM	92.3 M	0.9245	0.8396	0.9182	0.5259	231.2
**Improvement**	**−20.8 M**	**+3.22%**	**−0.16%**	**+1.72%**	**+3.08%**	**−42.2**
YOLOv7-W6 [20]	70.4 M	0.8211	0.8474	0.8905	0.4831	360
Pix2Pix + YOLOv7-W6	91.2 M	0.8588	0.8595	0.914	0.497	402.2
**Improvement**	**−20.8 M**	**+3.77%**	**+1.21%**	**+2.35%**	**+1.39%**	**−42.2**
YOLOv7-E6E [20]	151.7 M	0.8441	0.8451	0.8596	0.4914	843.2
Pix2Pix + YOLOv7-E6E	172.5 M	0.8654	0.8326	0.8641	0.4945	884.4
**Improvement**	**−20.8 M**	**+2.13%**	**−1.25%**	**+0.45%**	**+0.31%**	**−42.2**

## Data Availability

M. Lamane—AWR2944 mmWave Radar Dataset for YOLOv7. Available online: https://kaggle.com/datasets/a0a79ed59cce71bb788a634d130b5daf1d66b0c6f98ce34a5f9f46a24e02f5d6.
